# Dynamic polarization of tumor-associated macrophages and their interaction with intratumoral T cells in an inflamed tumor microenvironment: from mechanistic insights to therapeutic opportunities

**DOI:** 10.3389/fimmu.2023.1160340

**Published:** 2023-05-12

**Authors:** Jiashu Han, Luochu Dong, Mengwei Wu, Fei Ma

**Affiliations:** ^1^ 4+4 Medical Doctor Program, Chinese Academy of Medical Sciences and Peking Union Medical College, Dongcheng, Beijing, China; ^2^ Department of General Surgery, Peking Union Medical College Hospital (CAMS), Beijing, China; ^3^ Center for National Cancer, Cancer Hospital, Chinese Academy of Medical Sciences and Peking Union Medical College, Beijing, China

**Keywords:** immunotherapy, tumor-associated macrophages, tumor microenvironment, inflammation, immunometabolic dysregulation, macrophage polarization

## Abstract

Immunotherapy has brought a paradigm shift in the treatment of tumors in recent decades. However, a significant proportion of patients remain unresponsive, largely due to the immunosuppressive tumor microenvironment (TME). Tumor-associated macrophages (TAMs) play crucial roles in shaping the TME by exhibiting dual identities as both mediators and responders of inflammation. TAMs closely interact with intratumoral T cells, regulating their infiltration, activation, expansion, effector function, and exhaustion through multiple secretory and surface factors. Nevertheless, the heterogeneous and plastic nature of TAMs renders the targeting of any of these factors alone inadequate and poses significant challenges for mechanistic studies and clinical translation of corresponding therapies. In this review, we present a comprehensive summary of the mechanisms by which TAMs dynamically polarize to influence intratumoral T cells, with a focus on their interaction with other TME cells and metabolic competition. For each mechanism, we also discuss relevant therapeutic opportunities, including non-specific and targeted approaches in combination with checkpoint inhibitors and cellular therapies. Our ultimate goal is to develop macrophage-centered therapies that can fine-tune tumor inflammation and empower immunotherapy.

## 1 Introduction

**Figure 1:**
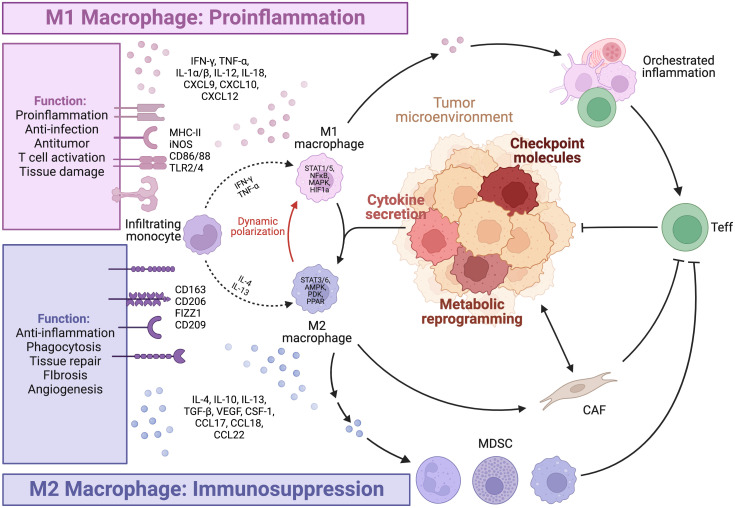
Macrophage polarization. The figure is created with BioRender.com.

Tumor-associated macrophages (TAMs) represent the most abundant and heterogeneous cell population in the tumor microenvironment (TME). The M0/M1/M2 model has been widely adopted to describe their broad spectrum of phenotypes and functions (**Figure 1**). Briefly, Toll-like receptors (TLRs) and type 1 cytokines stimulate the pro-inflammatory M1 phenotype. In contrast, alternatively activated M2 macrophages are further classified into subtypes including M2a, M2b, M2c, and M2d. Anti-inflammatory M2a macrophages with characteristic CD206 and TGF-β expression are induced by interleukin (IL)-4 and IL-13. M2b is closely associated with type 2 immunity and T helper 2 differentiation in response to parasitic and fungal infections. Glucocorticoids promote the deactivation of M2c macrophages (1). In the TME, tumor-associated factors such as adenosine favor the differentiation of M2d macrophages with high expression of IL-10 and VEGF (2). *In vitro* stimulation of cell lines under controlled experimental conditions often leads to highly reproducible results in terms of the aforementioned markers. However, these results are frequently disjointed with real-world situations.

The emergence of high-throughput and multi-omic technologies has led to advancements in the understanding of TAMs in patient samples, revealing the heterogeneity and plasticity of TAMs, particularly in the TME. TAM subtypes are dynamic spectrums, rather than fixed states of terminal differentiation, influenced by both ontogeny and environmental factors. This complexity exhibits patterns, as large-scale pan-cancer studies at the transcriptomic level have identified seven subtypes of TAMs that are conserved across multiple tumor types. These subtypes include the interferon-primed, immune regulatory, inflammatory cytokine-enriched, lipid-associated, pro-angiogenic, resident-tissue-macrophage-like, and proliferating subtypes (3). While such phenotype-based annotation is informative, it may not provide a complete understanding of the functional significance of these TAMs subtypes in tumor progression and immune activation.

**Figure 2:**
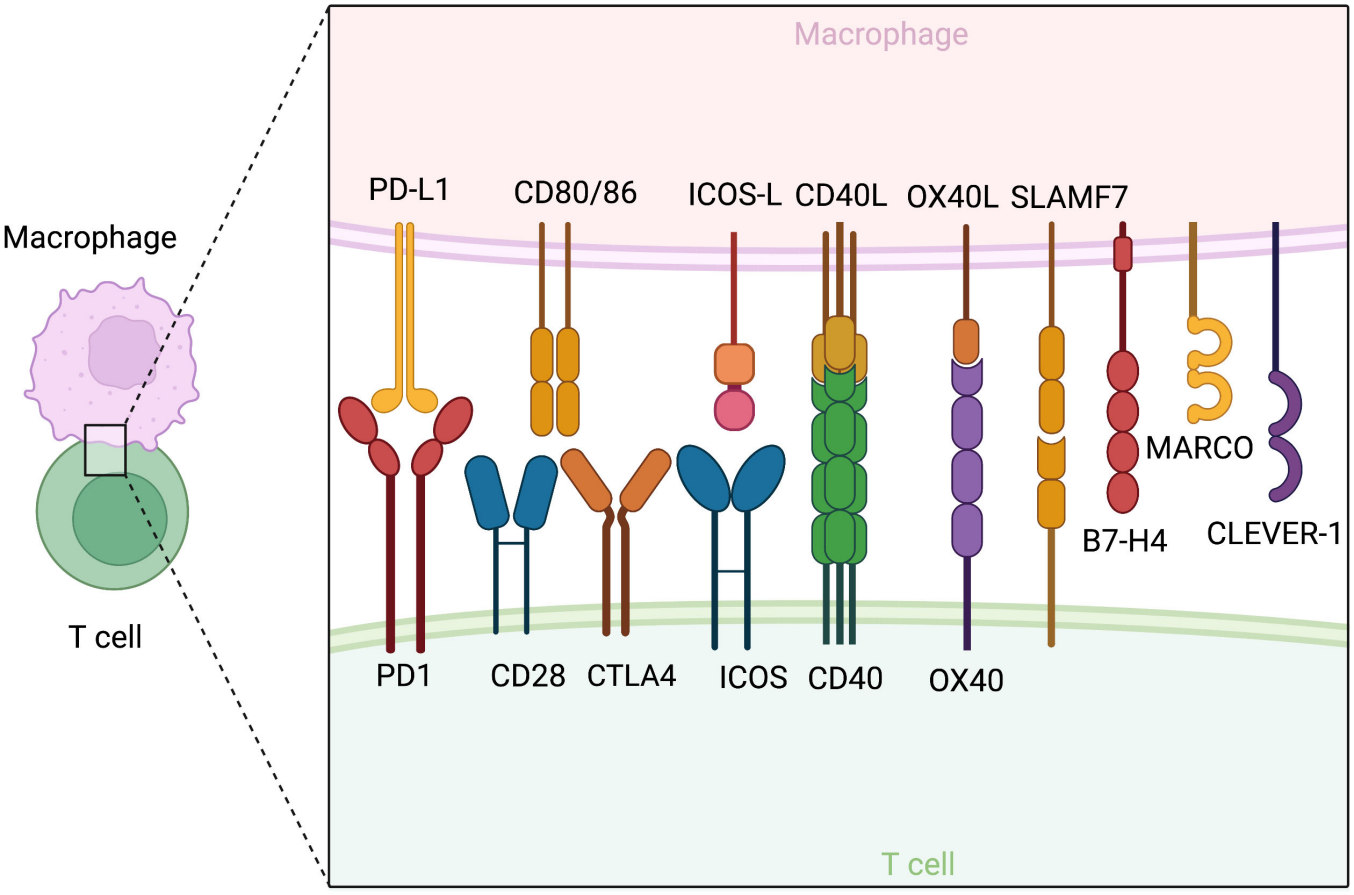
Co-stimulatory and co-inhibitory molecules on macrophages and their corresponding receptors on T cells. The figure is created with BioRender.com.

TAMs should be redefined in functional classes that exhibit either tumor-promoting or tumor-suppressive effects. Multiple factors, particularly those with similar functional effects, are under orchestrated regulation and exhibit patterns of co-expression. Antigen presentation, costimulatory molecules and activating cytokines, including IFN-γ, TNF-α, and IL-2, are needed for synergistic amplification of the immune response cascade (4). On the other hand, M2 cytokines (5) such as IL-4, IL-13, TGF-β, and IL-10, and inhibitory ligands collectively establish feedback loops where regulatory T cell (Treg)-secreted IL-13 stimulates M2 to secrete IL-10, further promoting Treg differentiation (6). Notably, phenotypic markers and function can be disjointed. CD206^+^ M2 TAMs conventionally considered to be tumor-promoting have been shown to be capable of antigen cross-presentation, stimulation of antitumor immune responses, and tumor regression in mouse models of melanoma and colorectal cancer, due to concurrent CD80 expression (7). In contrast, M1 alveolar macrophages without CD80/86 expression failed to present antigens effectively (8). Considering the importance of evaluating the functional identity of TAMs, we will further highlight several key pro-inflammatory and immunosuppressive factors (**Figure 2**).

## 2 Pro-inflammatory

### 2.1 TNF-α

Tumor necrosis factor-alpha (TNF-α) is an inflammatory cytokine produced in large amounts by macrophages upon pattern recognition receptor (PRR) activation, and is considered a key antitumor agent responsible for the suppression of tumor growth by activated macrophages (9–12). However, chronic exposure to TNF-α in a detrimental inflammatory context has pro-tumor functions, including angiogenesis, metastasis, T cell apoptosis, and exhaustion (13–15). Therefore, both activators and inhibitors of TNF-α have been attempted in clinical use and will be discussed later in the Therapeutics section.

### 2.2 IFN-γ

Interferon-gamma (IFN-γ), plays a crucial role in innate stimulation, antigen presentation, T cell activation, and effector function. While activated T and natural killer (NK) cells are the major sources of IFN-γ, TAMs also produce this cytokine (16–21). IFN-γ production is under intricate regulation, as it is enhanced by NFκB activation downstream of IL-12 and IL-18 stimuli (22–25) and STAT4 phosphorylation (25), but inhibited by IL-27 treatment (22). Studies in CAR-T and anti-PDL1 antibody therapies, as well as a mouse model of bladder cancer, have demonstrated that antitumor TAMs rely on both antigen presentation and IFN-γ secretion to activate CD4+ T cells. These CD4+ T cells then secrete IFN-γ to stimulate M1 polarization, establishing reciprocal amplification that sets the antitumor immune response in motion (26, 27).

### 2.3 MHC-II

Macrophages are capable of processing and loading antigens onto MHC-II, whose variable regions bind to specific antigenic peptides recognizable by their corresponding T cell receptor (TCR). Formation of this complex leads to TCR activation as the first signal of T cell activation. T cells are often localized in TAM-rich regions due to the ability of TAMs to present tumor-associated antigens and mediate T cell chemotaxis. However, impaired immunological synapses between TAMs and T cells can lead to T cell anergy and notably forming M2-Treg interactions, even in tumor-draining lymph nodes (28–30). Furthermore, prolonged interaction between TAMs and CD8+ T cells is a significant factor of T cell exhaustion (31) and can impede T cell infiltration (32).

### 2.4 Co-stimulatory molecules

CD80 (B7-1) and CD86 (B7-2) are classical M1 markers and serve as ligands for both the co-stimulatory CD28 and inhibitory CTLA-4 of the B7 family expressed by T cells. The expression level and density of CD80/86 determine their effects. Low-level expression favors CTLA4 binding and immunosuppression, while only high-density expression can effectively stimulate T activation in models of colorectal cancer (33). A study in dendritic cells revealed a transcription factor, PU.1, with the ability to bind to the CD80/86 promoter and induce transcription (34). The expression of CD80/86 is temporally dynamic, peaking after 24-48 hours of culture and decreasing at 60 hours, in line with the timeframe of macrophage exhaustion and deactivation (35). In addition to CD80/86, other costimulatory molecules, such as the ICOS, OX40, 41BB, and CD40 signaling axis, are induced by primary activating signals (36–42). Many therapeutic attempts have been developed based on these checkpoints, which will be discussed in the Therapeutics section.

## 3 Immunosuppressive

### 3.1 VEGF

Vascular endothelial growth factors (VEGFs) have well-characterized functions in promoting angiogenesis and TME remodeling. However, in addition to these functions, VEGF secreted by TAMs has been shown to exhibit strong immunosuppressive effects through autocrine signaling that favors M2 polarization and upregulates PD-L1 expression (43). Interestingly, VEGF-A and VEGF-C are considered crucial for angiogenesis and lymphogenesis, respectively (44, 45), while the expression of latter by perivascular TAMs helps contain lung metastasis (46). By selectively modulating these processes, it may be possible to promote good angiogenesis and lymphogenesis over the undesirable angiogenesis, thereby converting immunologically “cold,” poorly perfused tumors to “hot” tumors with increased immune infiltration and better responses to immunotherapies, all without increasing tumor metastasis.

### 3.2 TGF-β

Transforming growth factor-beta (TGF-β) is a M2 cytokine with crucial roles in normal physiology, mediating inflammation resolution and apoptotic clearance (47–50). TGF-β secreted by TAMs shapes the TME by monitoring immune cell statuses and exerting immunosuppressive functions (51). Both TAMs and tumor cells share the downstream pathway of TGF-β/SNAIL (52–55). In tumor cells, TGF-β signaling induces thrombospondin-1 (TSP1) secretion and Treg differentiation (56). In TAMs, TGF-β exhibits potent pro-M2 functions and promotes its own expression in an immunosuppressive feedback loop (52, 57–59). However, studies in injury and inflammatory bowel disease models suggest that the transient expression and activation of SNAIL is essential for macrophage recruitment to their site of function by affecting chemotaxis and motility (60).

### 3.3 Co-inhibitory molecules

TAMs, rather than tumor cells, are the primary source of PD-L1 with CD8 suppressive functions and a key driver of response to anti-PD1/PDL1 therapy (61–70). However, TAM PD-L1 expression may result from a reactive response to various inflammatory stimuli in an immunologically “hot” tumor (65), such as GM-CSF (71), S100A8/TLR4/MyD88 (69, 72), IL10 and IL-27 (73), IL32/PFKFB3 (74), TGF-β/PKM2 (75), and classical M1 TNF-α/NFkB and MAPK (73, 76–78) through the activation of STAT1 and, in particular, STAT3 (79, 80). TAMs are also known to take up tumor cell PD-L1 for expression (81). In addition to inhibiting T cell function, TAM PD-1 suppresses phagocytosis and impairs antigen presentation (82). However, it also mediates pro-inflammatory macrophage differentiation and secretion (83). Apart from PD-L1, several other molecules have been identified as potential targets for fine-tuning the immunomodulatory functions of TAMs. SLAMF7 and VISTA/PSGL1 are novel checkpoint molecules expressed by TAMs to drive T cell exhaustion (84–86). CLEVER-1 and B7-H4 have been identified as specific to suppressive TAMs correlated with dysfunctional cytokine production and T cell dysfunction (87, 88).

## 4 TME remodeling

### 4.1 Fibroblast

**Figure 3:**
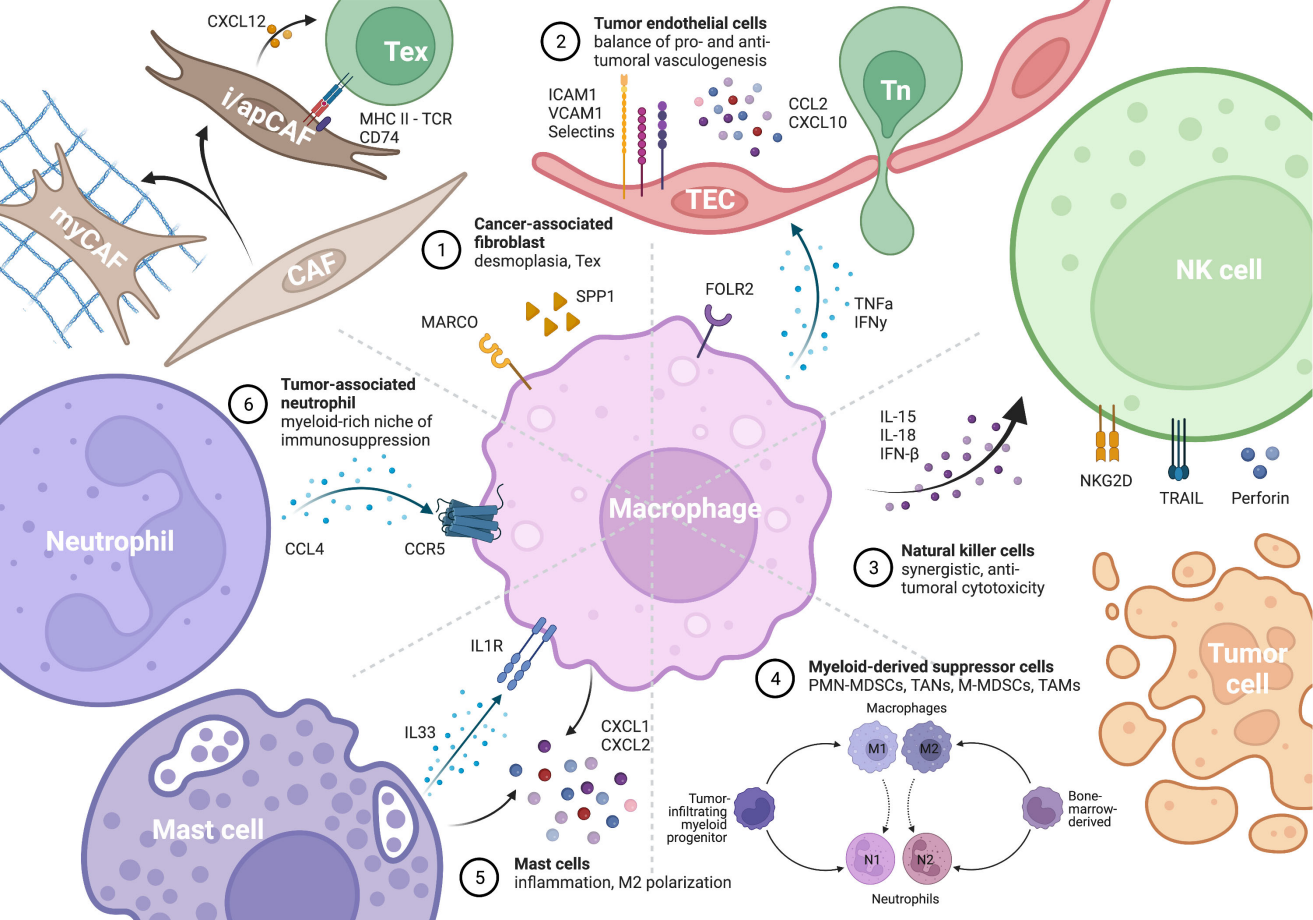
Macrophage as the key remodeler of TME inflammation by interacting with different cells. The figure is created with BioRender.com.

Cancer-associated fibroblasts (CAFs) are mesenchymal cells that undergo reshaping by the TME to achieve activation, differentiation, metabolic and epigenetic programming. CAFs display a high level of heterogeneity and dynamism and can be classified into three major subtypes with different capacities in stromal remodeling and inflammatory modulation, including inflammatory CAFs (iCAFs), myofibroblastic CAFs (myCAFs), and antigen-presenting CAFs (apCAFs) (89, 90). Despite their heterogeneity, CAFs are widely regarded as pro-tumorigenic and immunosuppressive cells that hinder immune cell infiltration, impair T cell activation and effector function, and promote T cell exhaustion. As the two primary components of the tumor stroma, TAMs and CAFs are extensively co-localized, collaborating to promote immunosuppressive desmoplasia (91) (**Figure 3**).

#### 4.1.1 T cell suppression through ECM remodeling

TAMs and myCAFs work together in extracellular matrix (ECM) deposition and remodeling, creating a desmoplastic microenvironment that acts as a barrier to T cell infiltration. A subset of TAMs expressing SPP1, also known as osteopontin (OPN), has been identified as markers of poor prognosis and immune checkpoint blockade (ICB) resistance in several types of tumors, including lung adenocarcinoma, hepatocellular carcinoma, and colorectal cancer (92–100). These SPP1+ TAMs are also characterized by high expression of the S100 family proteins, ECM-associated genes, and lipid metabolism features (101) and are often regarded as pro-inflammatory M1 macrophages. However, due to their low MHC-II expression and lack of co-stimulatory molecules, their antigen presentation capability is impaired, resulting in futile inflammation that fails to stimulate T cells effectively. Instead, SPP1+ TAMs interact with FAP+ CAFs to activate ECM deposition and inflammatory desmoplasia through SDC2 and MMP-2 interaction (94), IL-1, TGF-β (93), and CSF-induced granulin (102). Similar pro-fibrotic functions have also been reported in lung (103) and liver fibrosis (101, 104). Further studies have shown that the expression of SPP1 is induced by IL-17 or type 3 inflammation (104), metabolic reprogramming such as HIF-1 (93) and PGC-1α (105), and contact with tumor cells (106) or chemerin+ TGFβ+ CAFs (93). *In vitro* experiments in a mouse lung adenocarcinoma model have suggested that SPP1 mediates M2 polarization and the expression of IL-10, Arg1, and PD-L1, ultimately inhibiting CD4 T cells (106).

Much effort aimed at alleviating the immunosuppressive effects of the ECM (107, 108). The ECM provides not only integrin cross-talk (109) but also structural-biological coupling of mechanical sensing and signal transduction (110). Stiffness has extensive effects on T cell activation and antigen reactivity, cellular processes that are highly dependent on the spatial arrangement and proximity of receptors (111). High-density matrix hinders T cell proliferation and effector functions, favoring an immunosuppressive high ratio of CD4+/CD8+T cells (112), potentially through metabolic reprogramming (113). TAMs are a major source of matrix-remodeling enzymes: TAM-derived lysyl hydroxylase and lysyl oxidase mediate collagen cross-linking, matrix stiffening, and worse prognosis in breast cancer (114). The boundary between matrix-remodeling stromal cells has been further blurred by the discovery of SMAD3-dependent macrophage-myCAF transition (115).

#### 4.1.2 Direct suppression of T cells

TAMs can activate CAFs, resulting in the direct suppression of T cells. In pancreatic cancer, FAPα+ IL-6+ iCAF, TAMs and T cells form “reactive areas”, which inhibit T cell proliferation *via* both contact-dependent PD-L1 and PD-L2 mediated T cell exhaustion, as well as contact-independent PGE2 secretion (116–118). This ultimately leads to poor patient survival. In triple-negative breast cancer, CXCL12+ iCAF-induced T cell dysfunction is evidenced by a decoupling between survival benefit and T cell infiltration (119). In esophageal cancer, FGF2+ iCAF upregulates SPRY1 expression in T cells, a potent transcription factor for T cell exhaustion (120).

Recent studies have reported the presence of a rare subset of MHC-II+ CD74+ apCAFs in pancreatic (121–123), lung (118), breast, and colorectal (124) cancer, but not in prostate cancer. MSLN+ apCAFs from pancreatic cancer lack co-stimulatory molecules and can activate CD4+ T cells in an antigen-specific fashion to promote Treg differentiation. Targeted depletion of these cells is a major mechanism behind the therapeutic effect of anti-MSLN antibodies (122). In lung cancer, apCAFs express co-inhibitory molecules (CD73, IL-6, and IL-27) under the stimulation of CD39+ exhausted T cells, thereby creating a negative feedback loop of T cell exhaustion (125). Additionally, conditioned-medium from colorectal cancer cells up-regulates CTSS and immunosuppressive antigen cross-presentation in apCAFs (124).

Targeting these reciprocal interactions between TAMs, CAFs, and T cells to break the detrimental feedback loops may produce leveraged effects in alleviating immunosuppression (126). Overall, a deeper understanding of the complex interplay between TAMs, CAFs, and T cells in the TME could lead to novel therapeutic approaches for cancer treatment.

### 4.2 TEC function and angiogenesis

Endothelial cells play a crucial role in initiating immune responses and facilitating T cell trafficking. The three-step process of T-cell adhesion, extravasation, and infiltration relies on the expression of cell-cell interaction molecules such as ICAM-1, VCAM-1, and E/P-selectin, as well as secreted factors including CCL2 and CXCL10, by activated endothelial cells (127–129). However, tumor-associated endothelial cells (TECs) are reprogrammed by the TME into the first line of defense against incoming T cells (**Figure 3**). Secretions from TAMs, particularly the well-studied TNF-α and VEGF, play a critical role in determining the success or failure of angiogenesis (130).

#### 4.2.1 T cell recruitment by endothelial cells

TAMs can promote “good angiogenesis,” as TAM-dependent pro-inflammatory angiogenesis forms reactive areas of TAMs, T cells, and tumor endothelial cells (TECs) in pancreatic adenocarcinoma. TAMs are the major source of TNF-α in the TME, which activates TECs to allow for immunosurveillance (15, 131, 132). Inadequate TNF-α stimulation has been reported to reduce the expression of ICAM-1 on TECs, leading to impaired survival in patients with gut microbiota dysbiosis (133, 134). IFN-γ stimulates antigen presentation by tumor-associated lymphoid endothelial cells (135). Moreover, high endothelial venules (HEVs), which are essential for immune cell entry and ICB response, are supported by a specialized perivascular niche of enriched TAMs and sialomucin+ E/P-selectin+ TECs (136, 137). Such arrangements have been shown to potently attract CD8 T cell chemotaxis in cerebral malaria (138) and tumors, leading to markedly perivascular primed CD8 T cells and better survival in breast cancer (139).

#### 4.2.2 Pro-metastatic angiogenesis

On the other hand, TAMs are also the major source of VEGF in the TME. The combined use of anti-VEGFR and ICB has achieved impressive success in multiple clinical settings (140). VEGF stimulation leads to the development of immunosuppressive tumor endothelial cells (TECs) expressing GPNMB (141) and PD-L1 (142) in hepatocellular carcinoma and melanoma, respectively, resulting in T cell exhaustion. The combination of a VEGF inhibitor and ICB led to high endothelial venule (HEV) formation and T cell infiltration (143). Specific delivery of LIGHT to tumor vessels through vascular targeting peptide (VTP), known as the LIGHT-VTP therapy, potently induced tertiary lymphoid structures (144). Macrophages, which naturally express LIGHT in adipose inflammatory responses (145) and atherogenesis (146), are promising targets for the induction and amplification of LIGHT expression.

TAMs are the key to promoting good angiogenesis over immunosuppressive and pro-metastatic angiogenesis (147, 148). Specific targeting of VEGF+ TAM subsets responsible for immunosuppressive angiogenesis, while sparing the T cell attractant FOLR2+ and peri-HEV TAMs, may improve the TME and sensitize the tumor to ICB treatment (149).

### 4.3 Crosstalks with other immune cells

TAMs actively interact with other immune cells through surface molecules and cytokine secretions. These powerful engines are central to the formation, amplification and maintenance of inflammation in the TME. Such activities warrant antitumor immune response, but also end up in inflammation-mediated immunosuppression and exhaustion. The concept of tertiary lymphoid structures (TLS) refers to organized clusters of immune cells, including TAMs, dendritic cells, B cells, and T cells, which play a crucial role in refreshing adaptive immunity (150, 151). In this context, TAMs act as amplifiers and sustainers of inflammation by engaging in reciprocal interactions with other immune cells, including the recruitment of B cells, which produce IgG to further activate TAMs (152). Interestingly, TAMs and TLS exhibit different prognostic values across different types of cancer: while they are favorable in pancreatic and hepatocellular cancer, they are considered hazardous in breast and colorectal cancer (153). This suggests that the effects of inflammation are context-dependent: TAMs have the potential to promote immune cell infiltration into immunologically “cold” tumors, but for tumors challenged by chronic inflammation, inhibiting sustained TAM activation may alleviate inflammatory fibrosis and T cell exhaustion (154).

#### 4.3.1 NK cells

As part of the innate immune system, the activation and effector function of NK cells depend on a balance of inhibitory and stimulatory signals, allowing them to recognize and kill MHC-I deficient tumor cells, and at the same time rendering them susceptible to modulation by macrophages (155) (**Figure 3**). The cooperation between M1 macrophages and NK cells is crucial for effective immune response in both infection and tumor settings (156–158). Mechanistically, cytokines produced by M1 macrophages, such as IL-15, IL-18, and IFN-β, can upregulate NKG2D expression on NK cells and enhance their cytotoxic activity (159). However, in the TME, tumor cells and VCAM1+ CAFs can polarize macrophages towards an M2 phenotype, indirectly suppressing NK cell activity and promoting immune evasion (160, 161).

#### 4.3.2 Neutrophils

Myeloid-derived suppressor cells (MDSCs) are a heterogeneous population of cells of myeloid origin that are capable of potently suppressing T and NK cells. This population is often immature and exists in various states of differentiation, making consensus classification and therapeutic development challenging. MDSCs can be broadly classified into two major groups: polymorphonuclear (PMN-MDSC) and monocytic (M-MDSC) (**Figure 3**). Unlike TAMs or tumor-associated neutrophils (TANs), which refer to macrophages and neutrophils infiltrating into the TME, MDSCs are derived from the bone marrow under the remote influence of tumors and can be found outside the TME in peripheral blood and spleen. Despite differences in origin and cellular markers, MDSCs share many similarities in effector functions and extensivel cross-talks (162, 163). M-MDSCs and TAMs have been reported to be more potent in immunosuppression than PMN-MDSCs and TANs, although neutrophils usually outnumber macrophages in the TME (164). The myeloid-rich immunosuppressive landscape in liver, stomach, and breast cancer is constituted by reciprocal induction and synergistic action between CCR5+ TAMs and CCL4+ TANs (164–168). However, mutual exclusion of the two species has also been reported in breast cancer, with TANs being the more resistant group to ICB (169).

#### 4.3.3 Mast cells

Mast cells, a type of myeloid cell with specialized granules containing histamine and heparin, have been relatively overlooked in tumor immunology despite their importance in immune surveillance of normal tissue. In lung cancer, the formation of TAM-mast cell islets allows for mutually enhancing, synergized CXCL1/2 secretion, which has potent immune attracting and antitumor effects that are beneficial for survival (170–172). However, mast cells have been shown to exert pro-tumor functions in prostate, stomach, and pancreatic cancers, mechanistically through IL33-mediated M2 polarization (173–176). A deeper understanding of the dual roles of TAM-mast cell interaction in the TME may reveal novel therapeutic opportunities (**Figure 3**).

## 5 Metabolism

### 5.1 Glucose metabolism

**Figure 4:**
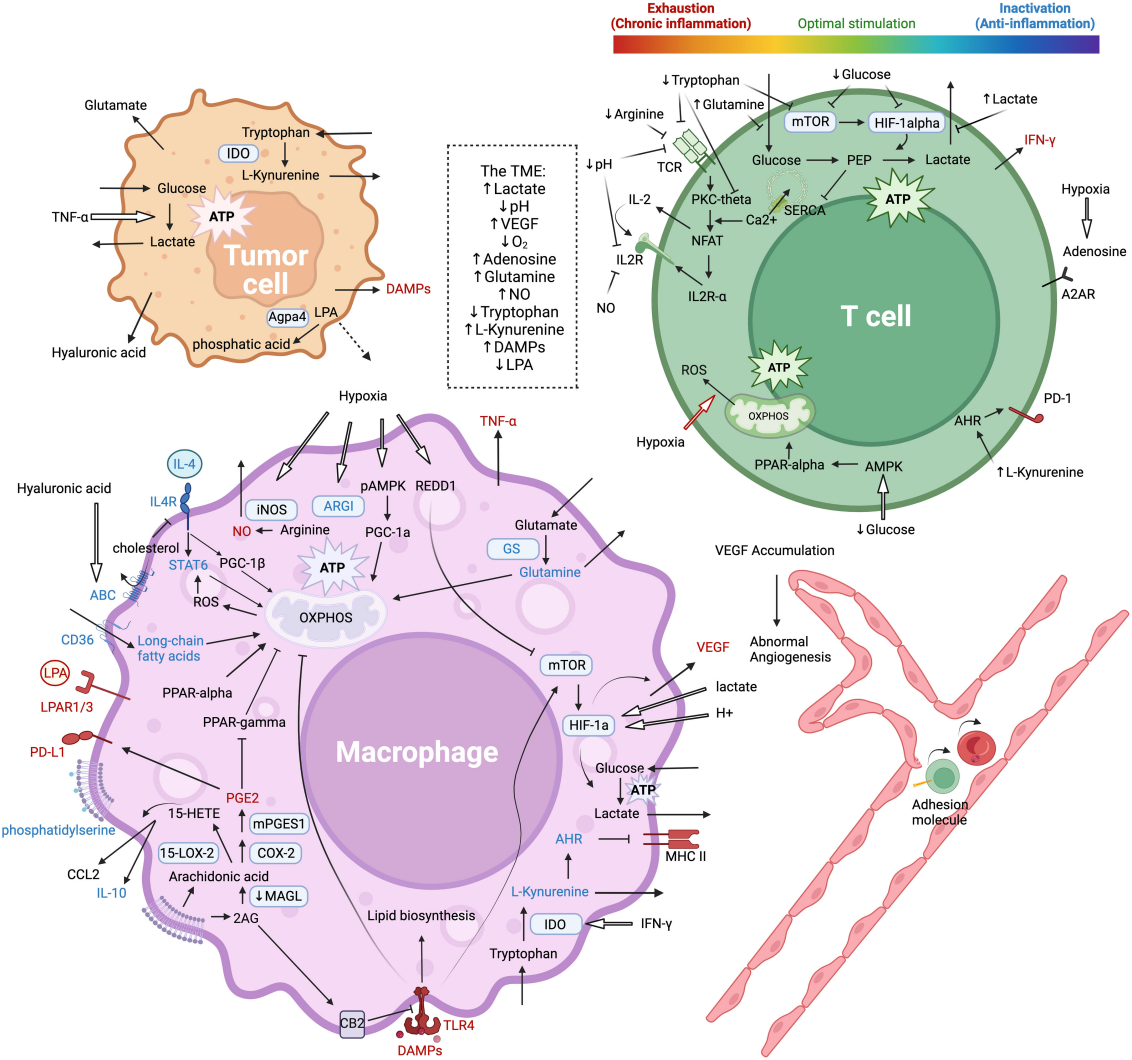
Metabolic interplay between macrophages, tumor cells, and T cells, and their effects on the inflammatory state of the TME and on T cell functions. Molecules in red and blue are clear pro-inflammatory and anti-inflammatory mediators respectively. The figure is created with BioRender.com.

Tumor cells primarily rely on aerobic glycolysis to produce energy, leading to the accumulation of lactate, acidic pH, and limited glucose availability in a metabolically hostile microenvironment (177, 178). Altered pathway activities, such as the key pathways AMPK/PGC1, HIF-1α, AKT/mTOR, and MAPK, lead to profound changes in metabolic preferences between glycolysis and oxidative phosphorylation. Subsequent metabolic adaptations give rise to phenotypic and functional outcomes in terms of pro- and anti-inflammatory activities, as summarized in the introduction section. These intricately regulated processes are not specific to any particular cell type but often share common molecular mechanisms across diverse cellular contexts, resulting in context-specific consequences. These phenotype-function correlations highlight the importance of master metabolic regulators and the crosstalk between pathways (**Figure 4**).

#### 5.1.1 TAMs

Different signaling pathways and metabolic programs can independently upregulate either M1 or M2 specific characteristics without affecting each other, indicating a mutually-independent regulation mechanism (179, 180). Signaling through HIF-1α, AKT/mTOR, and MAPK pathways promotes aerobic glycolysis and converge on the downstream effector JAK/STAT, leading to subsequent M1 features, while the stress-responsive AMPK/PGC1 pathway and PDK signaling promotes mitochondrial function and oxidative phosphorylation, leading to M2 polarization (52, 65, 179, 181–196).

However, single cell sequencing studies have identified anti-inflammatory TAMs that perform both glycolysis and oxidative phosphorylation (197). Also, hypoxia also lead to increased production of M2 secretions by TAMs, especially VEGF and TGF-β, as yin and yang to keep immune reactions in check (47, 198, 199). However, such mechanisms in the TME exacerbate glucose and oxygen deficiency and further metabolic challenges (183, 187, 200–204). Interestingly, HIF-2α has the opposite role and stimulates the production of secreted VEGF receptor (sVEGFR-1) to neutralize the biological activity of VEGF-A, reducing angiogenesis and tumor growth (205–207). However, the effects of some stimuli are dependent on the baseline activation status of TAMs, leading to differing outcomes between M1 and M2 (47, 208). The response to external stimuli is coupled to finely regulated signaling pathways, with multiple targetable points and well-developed activators and inhibitors. Therefore, understanding the links between these pathway activities, metabolic states, and functional phenotypes in TAMs would guide the development of TAM-centered metabolic regulators to modulate the inflammatory state of the TME (181, 191, 209, 210).

#### 5.1.2 T cells

High oxygen consumption in the oxidative phosphorylation process of TAMs in the TME can lead to hypoxia, which inhibits the function of T cells in several ways. The hypoxic TME leads to abnormal angiogenesis and dysfunctional endothelial cells that express lower levels of cell adhesion molecules, making it difficult for T cells to attach and infiltrate (134). Adenosine accumulation activates the inhibitory A2A receptor on CD8+ T cells (211, 212). Hypoxia also leads to long-lasting interactions between TAMs and CD8+ T cells, eventually causing T cell exhaustion (31). The acidic pH of a hypoxic TME decreases the expression of TCR and IL-2Ra and leads to T cell inhibition, reversible with proton pump inhibitors (213). Mechanistically, lactate accumulation in the extracellular compartment directly prevents the secretion of lactate from T cells, driving the glycolysis equilibrium to the left and hindering T cell functions (214).

Upon activation, T cells switch from oxidative phosphorylation to aerobic glycolysis to meet the high energy demands while minimizing reactive oxygen species (ROS) production (215). However, in response to the metabolic challenges of TME, activated T cells preferentially utilize the AMPK/PGC1 pathway instead of the HIF-1α and PAM pathways, suppressing glycolysis (216). Subsequent ROS overproduction and mitochondrial stress lead to T-cell exhaustion (217), while decreased availability of a key metabolite phosphoenolpyruvate alleviates SERCA inhibition to disrupt Ca2+-dependent NFAT signaling and impair effector function (218). Despite these detrimental effects of oxidative phosphorylation, it is also essential for the viability and activity of T cells. PPAR-α improves the metabolic competitiveness of T cells, indicating the importance of a balanced metabolic state for T cell function (216, 219)

### 5.2 Amino acid metabolism

#### 5.2.1 Glutamine

TAMs express glutamine synthetase (GS), an important enzyme in the conversion of glutamate secreted by tumors to glutamine, which is then released back into the TME as a building block for tumor cells (189, 220–222). This process not only supports tumor anabolism but also affects TAM polarization. Inhibiting GS and allowing succinate to accumulate promotes HIF-1α signaling, resulting in increased glucose flux through glycolysis and the pro-inflammatory M1 phenotype, restoring the proliferation of co-cultured T cells and promoting good angiogenesis *in vivo* (189, 223). Additionally, high levels of glutamine in the TME directly suppress T cells, possibly by competing with glucose transporters and suppressing glycolysis (224).

#### 5.2.2 Arginine

Under metabolic challenges of the TME, TAMs can be polarized to preferentially express arginase-1 (ARG1) or inducible nitric oxide synthase (iNOS), resulting in different functional phenotypes (200, 225). iNOS-produced nitric oxide (NO) is a pro-inflammatory product of M1 phenotype, whereas ARG1 converts arginine into ornithine and urea, making it an immunosuppressive M2 marker due to competition with iNOS for the limited arginine pool (226–228). High expression of the arginine transporter and ARG1 in TAMs can deplete arginine from the TME, leading to the loss of the zeta-chain of CD3 in activated T cells and impaired antitumor immune activity (228). Surprisingly, arginine-depletion therapy paradoxically increases CD8+ T cells and pro-inflammatory TAMs in the TME, possibly due to the fact that T cells expressing argininosuccinate synthase 1 (ASS1) can synthesize arginine from citrulline and succinate, whereas ARG1+ TAMs and tumor cells are more dependent on extrinsic arginine (229–231). Moreover, recent studies have shown that citrulline depletion by ASS1 activity in the urea cycle is important for the pro-inflammatory functions of macrophages, which could partly explain how arginine depletion may potentially help reprogram TAMs (232, 233). Thus, the competition for arginine between TAMs and T cells in the TME is a critical metabolic checkpoint that can influence the balance between immunosuppressive and inflammatory responses.

#### 5.2.3 Tryptophan

Tryptophan is an essential amino acid required by T cells. Its metabolism by indoleamine 2,3-dioxygenase (IDO) through the kynurenine pathway generates immunosuppressive catabolites, particularly kynurenine. The expression of IDO is induced by inflammatory stimuli to prevent excessive immune responses. However, this process is often exploited by tumors and immunosuppressive cells in the TME. TAMs interaction with specific CD69+CD8+ T cells enhances IFN-γ secretion to upregulate IDO in TAMs, creating a negative feedback loop to keep T cell activities in check (234–236).

Tryptophan starvation and catabolites decrease mTOR and PKC-θ signaling, resulting in reduced CD3ζ expression and cell cycle arrest in T cells (237–239). L-Kynurenine, the major kynurenine pathway metabolite, enters cells through transporters SLC7A8 and PAT4 to activate the aryl hydrocarbon receptor (AHR), a cytoplasmic transcription factor expressed in T cells and TAMs (240–242). In T cells, AHR activity inhibits cytokine secretion and promotes PD-1 expression (240). In TAMs, AHR lowers MHC-II expression but enhances interaction with regulatory T cells (241). Notably, L-Kynurenine is produced not only by IDO+ TAMs or tumor cells but also by microorganisms such as Lactobacillus from pancreatic cancer (243). In this case, a low-tryptophan diet is beneficial by increasing the number of cytotoxic CD8+ T cells in the TME (243).

### 5.3 Lipid metabolism

#### 5.3.1 Fatty acids

Pro-inflammatory TAMs are characterized by active lipid biosynthesis, which is coupled to the PAM pathway and glycolysis, as previously described in the section on glucose metabolism (191, 202). In contrast, fatty acid oxidation (FAO) is associated with other immunosuppressive and pro-M2 factors including oxidative phosphorylation, IL4/STAT6 signaling, AMPK/PGC1 pathways, and ROS activity (190, 192, 244, 245). The accumulation of long-chain unsaturated fatty acids, oxLDL, and lipid droplets in the lipid-enriched TME allows preferences of FAO over other methods of energy production (49, 246, 247). Active FAO depends on several important factors involved in lipid transport, metabolic enzymes, and regulatory molecules. The transcription factor PPAR-α drives the expression of FAO and OXPHOS genes (248). ABHD5 is a coactivator for HSL, the rate-limiting enzyme in triacylglycerol hydrolysis, and a stimulator of PPAR-α (249–252). APOE lipid transporter (253) and CD36 fatty acid translocase (49) both mediate the accumulation of intracellular fatty acids. The activity of NFκB and RIPK3 promotes the degradation of the aforementioned molecules and counters FAO (209). These molecules are often highly expressed in tumor cells and some TAM subsets, while downregulated in specific inflammatory TAMs, mediating differential reprogramming of lipid metabolism in a context-dependent fashion. Therefore, further research on these key molecules may enable the differentiation of TAMs from tumor cells and the cell-type-specific metabolic rewiring. Understanding the regulation and role of FAO in TAMs is critical as it can shape the tumor microenvironment and the immune response.

#### 5.3.2 Phospholipids and derivatives

Phospholipids and their derivatives are not only major components of the plasma membrane with structural functions, but also important bioactive molecules with potent signaling functions. Different species of phospholipids have distinct effects, whose production and metabolism are often harnessed by tumor cells and TAMs in the TME. For example, phosphatidylserine (PS) exposure on the outer plasma membrane is a key feature of apoptotic cells. Together with other “eat me” signals, PS promotes phagocytosis and TGF-β production, making it a targetable point to stimulate inflammation (254, 255). Lysophosphatidic acid (LPA) has dual effects depending on the available receptors. In models of colorectal cancer, secreted LPA by cancer cells is recognized by LPAR1-3 on TAMs, driving the expression of inflammatory genes. However, in the ascites of patients with ovarian cancer, LPA binding to LPAR5/6 is associated with M2 polarization, immunosuppression, tumor metastasis, and poor outcomes (256). AGPA4 expressed by cancer cells converts TME LPA to phosphatidic acid, undermining its pro-inflammatory effects (257). 15-LOX-2+ TAMs from renal cell carcinoma catalyze the degradation of arachidonic acid into 15-HETE, which stimulates CCL2 and IL-10 production to recruit immunosuppressive TAMs (258, 259). In murine bladder and prostate cancer, specific TAMs express mPGES1 and COX-2 to produce large amounts of prostaglandin E2 (PGE2), a molecule with well-investigated immunosuppressive effects through NFκB-mediated PD-1/PD-L1 expression in both TAMs and T cells, unleashing the inhibitory effect of PPAR-γ on fatty acid oxidation and oxidative phosphorylation, and increasing secretion of immunosuppressive factors especially VEGF (260–265). In colorectal cancer, tumor-induced downregulation of monoacylglycerol lipase (MAGL) in TAMs is associated with the accumulation of tri-, di-, and mono-glycerides along with arachidonoylglycerol, enhancing CB2 receptor activity to antagonize TLR4 signaling and mediate immunosuppression (266).

#### 5.3.3 Cholesterol

Cholesterol and its derivatives have been shown to have immunomodulatory effects. In the context of metastatic ovarian cancer, cholesterol is considered beneficial. Tumor-derived hyaluronic acid has been shown to stimulate TAMs to express ABC transporters, which facilitate cholesterol efflux and lipid raft depletion, leading to IL-4 signaling and immunosuppressive reprogramming (267, 268). The liver X receptor (LXR), which is activated by cholesterol derivatives, has been shown to favor the expression of pro-inflammatory genes in TAMs. Pharmacological manipulation of the cholesterol represents a promising strategy for reprogramming TAMs towards an anti-tumor phenotype (269, 270). However, in certain situations, the cholesterol family may also have immunosuppressive and pro-tumor effects.

## 6 Therapeutics

Therapeutic targeting of TAMs by antibody/cytokine administration or depletion is rather unspecific. However, recent drug developments attempted more precise approaches to achieve better efficacy and reduce off-target effects.

### 6.1 Polarization

Despite the phenotypical diversity of TAMs, the classical model of M1-inflammatory and M2-immunosuppressive TAMs remains a useful reference framework for the overall polarization direction, which is closely linked to the functional and phenotypic status of the TAMs.

#### 6.1.1 Chemotherapy, radiation therapy and TKI

Conventional therapies are generally considered immunostimulatory (271–276) by inducing cancer cell death and increasing the release of damage-associated molecular patterns (DAMPs) into the TME (277, 278). TAMs express several PRRs such as TLR4, which can recognize DAMPs and activate downstream signaling pathways including NF-kB and inflammasome, leading to phenotypical, metabolic and functional changes, and the expression of pro-inflammatory genes (191, 202, 279). The synergistic effect between conventional therapies and immunotherapies, with the former priming for the latter, leads to improved efficacy of combined therapy compared to monotherapy. However, the suboptimal effect and non-specificity, along with the exhausting effect of chronic futile inflammation, highlight the need for further development of regulated immune stimulation (280, 281).

#### 6.1.2 Innate activation

Numerous natural and synthetic compounds have been found to activate the innate immune system, making them potential therapeutic agents that are currently under active pre-clinical development and clinical translation (282). PRR agonists have shown promising results in preliminary clinical trials (283, 284), particularly when combined with other immunostimulatory agents. For example, the TLR4 agonist monophosphoryl lipid A (MPLA) has been combined with IFN-γ (285), and the TLR7/8 agonist has been linked to anti-HER2 to form PRR-antibody-drug conjugates (286) (**Table 1**). These agonists are capable of promoting M1 polarization and TME inflammation. STING agonists have also been widely used in pre-clinical and clinical studies. They are administered through direct intratumoral injection or nanoparticle-based intravenous administration to increase the specificity of delivery to tumor cells or TAMs (287, 288). Recently, a STING agonist derived from microbiota has been identified to be associated with improved response to ICB in mouse models, thus implicating it as a potential mechanism underlying the benefits observed in high-fat diet or fecal transplantation of human responder (289).

**Table 1:** Clinical trials of representative macrophage-centered therapies.

Name	Type	Combination	Trial Number	Phase	Cancer Type
BDC-1001	Anti-HER2 conjugated TLR 7/8 dual Agonist	**+/- Nivolumab**	NCT05091528	I/II	HER2 positive solid tumors
SBT6050	Anti-HER2 conjugated TLR 8 Agonist	**+/- Pembrolizumab/Cemiplimab**	NCT04278144	I	HER2 positive solid tumors
NIR178	A2AR Inhibitor	PDR001	NCT03207867	II	Different Solid Tumors
Ciforadenant	A2AR Inhibitor	Ipilimumab + Nivolumab	NCT05501054	I/II	Renal Cell Carcinoma
CB-839	GS Inhibitor	**+/- Everolimus**	NCT03163667	II	Clear Cell Renal Cell Carcinoma
CB-1158	ARGI Inhibitor	**+/- Pembrolizumab**	NCT02903914	I/II	Different Solid Tumors
Metformin	Respiratory Complex I Inhibitor	N/A	N/A	II	Esophageal Squamous Cell Carcinoma
TPST-1120	PPAR-Alpha Antagonist	**+/- Nivolumab**	NCT03829436	I	Different Solid Tumors
FP-1305	Anti-CLEVER-1 Antibody	N/A	NCT03733990	I/II	Different Tumors
GSK3359609	ICOS Agonist Antibody	Pembrolizumab + 5FU-Platinum	NCT04428333	II/III	Head and Neck Squamous Cell Carcinoma
JTX-2011	ICOS Agonist Antibody	**+/- Nivolumab/Ipilimumab/Pembrolizumab**	NCT02904226	I/II	Different Tumors
MEDI-570	ICOS Agonist Antibody	N/A	NCT02520791	I	Different Lymphomas
KY1044	ICOS Agonist Antibody	**+/- Atezolizumab**	NCT03829501	I/II	Different Solid Tumors
GSK3359609	ICOS Agonist Antibody	**+/- Pembrolizumab/Other Arms**	NCT02723955	I	Different Tumors
Magrolimab	Anti-CD47 Antibody	Cetuximab	NCT02953782	I/II	Sarcoma and Colorectal Cancer
TG-1801	Anti-CD47 Antibody	Ublituximab	NCT03804996	I	B-cell Lymphoma
MK-4830	Anti-ILT4 Antibody	**+/- Pembrolizumab/Other Arms**	NCT03564691	I	Different Tumors
CT-0508	CAR Macrophage Against HER2	Pembrolizumab	NCT04660929	I	HER2-Overexpressing Solid Tumors

Systemic administration of TNF-α has been limited by adverse reactions, as phase 1 studies have generally been disappointing due to sepsis-associated symptoms and dose-limiting toxicities with little or no favorable antitumor activity (290–295). Intratumoral administration or delivery into specific arteries (isolated limb or hepatic perfusion) has achieved some therapeutic effects in selected tumors such as Kaposi’s sarcoma (296), high-grade soft tissue sarcoma (297), and liver cancer (298, 299). More targeted delivery into the TME *via* engineered malignant cell homing has improved response in mouse models of breast cancer and melanoma (300). TAMs can also be engineered to produce inflammatory cytokines under specific TME conditions, with the IFN-γ gene construct controlled by a synthetic promoter inducible by hypoxia (HRE3x-Tk) (301). Interestingly, anti-TNF-α antibodies, which are extensively used in the treatment of autoimmune diseases, are also potentially applicable in the treatment of inflammatory tumors (302). The combined use of Infliximab and ICB, first to control ICB-related adverse effects such as colitis, did not impair antitumor effects but rather demonstrated synergistic effects and enhanced response in animal models, melanoma, and further clinical trials (303–306).

#### 6.1.3 CSF-1/CSF-1R

CSF1R, a receptor tyrosine kinase, plays a crucial role in the differentiation and maintenance of M2 TAMs. Many small molecules inhibitors and antagonistic antibodies against CSF-1R have been developed, among which PLX3397 (pexidartinib) has been FDA-approved for treating tenosynovial giant cell tumor in 2019 (307, 308). These agents serve as a valuable foundation for anti-TAM therapeutic strategies, exhibiting synergistic effects with chemotherapy, radiotherapy, and ICB. Anti-CSF-1R antibody is promising as an addition to reverse resistance to anti-VEGF therapy and taxane chemotherapy (309, 310). A study in pancreatic cancer demonstrated that CSF-1/CSF-1R blockade up-regulated PD-L1 and CTLA-4, justifying that the combination of TAM reprogramming therapy with ICB may yield maximum effect (311). However, the effect of CSF-1R-targeted TAM depletion in the context of the heterogeneous TME requires further investigation, as preferential depletion of inflammatory TAMs but sparing pro-angiogenic/tumorigenic TAMs may lead to unwanted effects (94).

#### 6.1.4 Others

CD206, an M2 macrophage marker, has been utilized as a guidepost for precise targeting of immunosuppressive TAMs (312). Various approaches such as nanoparticle-based mRNAs of IRF5, IKK-β, and miRNA-155, Fe3O4-based poly(lactic-co-glycolic) acid (PLGA) nanoparticles conjugated with anti-CD206, and RP-182 peptide, a small molecule inhibitor of CD206, have been developed (313–315). However, it is important to consider the heterogeneity and dynamic nature of TAMs, and to note that CD206 expression alone may not fully define immunosuppressive TAMs. Recent studies have shown that CD206-expressing TAMs are also capable of cross-presenting tumor-associated antigens to activate T cells (7), suggesting that CD206-directed therapies may inadvertently deplete beneficial TAMs. Advancements in high-throughput technologies have identified additional markers providing novel targets for intervention. For example, anti-MARCO-antibody and anti-Clever-1 antibody (FP-1305) were both capable of causing a phenotypic switch in TAMs from immunosuppressive to pro-inflammatory, and the combination of anti-Clever-1 antibody (FP-1305) with ICB showed synergistic benefits in aggressive tumors that were unresponsive to ICB (158, 316, 317).

### 6.2 Metabolism

The metabolic state of TAMs is closely linked to their phenotypical and functional polarization, as they both respond to and influence the inflammatory microenvironment. Many therapies have been developed targeting the metabolism of TAMs and their associated effects on T cells.

#### 6.2.1 A2AR antagonism

Both pro- and anti-inflammatory TAMs have been found to exacerbate TME hypoxia, leading to the accumulation of adenosine, which in turn acts on A2AR to suppress T cell activity (211, 212). A2AR antagonists have shown promising therapeutic responses in various pre-clinical studies, particularly in the setting of chimeric antigen receptor T (CAR-T) cell therapy (211, 318). Currently, several phase I/II clinical trials investigating A2AR inhibitors, some in combination with ICB, are ongoing (see **Table 1**).

#### 6.2.2 Amino acid metabolism

Targeting glutamate-glutamine metabolism (189, 319) with glutamine antagonists, GS blockade, and glutamine transporter inhibition, effectively drives M1 polarization and antitumor response, especially when used in combination with ICB (189, 224, 319, 320). A promising GS inhibitor, CB-839/Telaglenastat, is currently being evaluated in several phase I/II clinical trials. Preliminary of results have shown decreased mortality but increased incidence of serious adverse events when combined with the mTOR inhibitor Everolimus (**Table 1**). Moreover, glutamine antagonism has been found to reduce the expression of IDO in both tumor cells and TAMs, which suggests an intrinsic link between glutamine and tryptophan metabolism (319).

Restoring tryptophan availability and depleting kynurenine by inhibiting IDO has been an attractive therapeutic approach (239–241). However, the combination of the IDO inhibitor Epacadostat with Pembrolizumab failed to show antitumor activity in a phase II trial as compared to monotherapy (320). The lack of efficacy may be attributed to enzymes with redundant functions, such as IDO1, IDO2, and tryptophan 2,3-dioxygenase (TDO), indicating the need for dual- or pan-inhibitors.

The ARG1 inhibitor CB-1158 has demonstrated promising results in pre-clinical models, both as a monotherapy and in combination with ICB as well as adoptive T or NK cell therapy (226, 228, 321). A phase I/II clinical trial of CB-1158 monotherapy suggested good tolerability (**Table 1**), but paradoxically, better antitumor response was observed with CB-1158 monotherapy compared to combination with pembrolizumab. The reason behind the compromised effect of combinatorial therapy requires further investigation.

#### 6.2.3 Modulation of OXPHOS and lipid metabolism

OXPHOS and FAO are associated with immunosuppression in the TME (188). In preclinical models, the inhibition of FAO by etomoxir, a drug that targets the rate-limiting enzyme carnitine palmitoyl-transferase 1a (CPT1a), led to a reduction in tumor growth (190). Similarly, CD36 knockout had a similar effect, indicating the potential of CD36-based therapies for TAM-specific inhibition of OXPHOS. Conversely, the CB2 cannabinoid receptor has been shown to inhibit TLR4 signaling and promote fatty acid oxidation, and pharmacological antagonism of this receptor reduced tumor growth independently of CB2 expression in tumor cells (266).

Direct inhibition of the electron transport chain can also reduce OXPHOS in TAMs (322). The respiratory complex I inhibitor metformin, an anti-diabetic drug that has been repurposed to treat cancer, has shown promising results in preclinical studies (**Table 1**). In a phase II trial, low-dose metformin was able to reprogram an inflammatory TME by increasing the number of anti-tumor TAMs and CD8+ T cells infiltration while decreasing infiltration of Treg cells. However, there was no significant change in the growth or apoptosis markers of tumor cells (323). Future clinical trials that combine metformin with other therapeutics, particularly ICB and cellular therapy, may reveal any potential synergistic effects.

Phospholipids and cholesterol also play a crucial role in regulating TAM phenotypes (260–262, 266–268). Inhibitors of COX2 or antagonists of PGE2 receptors in combination with ICB have been shown to reprogram the TME and increase T cell infiltration (324). Additionally, in patients with colorectal cancer, treatment with the DNA methyltransferase (DNMT) inhibitor 5-aza-2’-deoxycytidine (5Aza) decreased cholesterol efflux from the ABC transporter of TAMs, leading to pro-inflammatory effects and improved function of CD4 and CD8+ T cells (325).

It is important to remember that most metabolic pathways and corresponding drugs lack cell type selectivity. Since tumor cells, TAMs, and T cells depend on similar non-specific metabolic pathways, targeting TAM glycolysis with such drugs may hinder the proliferation and activity of T cells and promote tumor growth simultaneously. For example, activated T cells generally rely on aerobic glycolysis, but in low-glucose and hypoxic conditions like the TME, OXPHOS is crucial for their survival and function. Animal models have shown that a PPAR-α agonist can stimulate OXPHOS and FAO, leading to increased CD8+ T cell cytotoxicity and enhanced efficacy of anti-PD1 (219). Conversely, the pro-M2 effects of PPAR-α on TAMs justify the use of PPAR-α antagonists in combination with nivolumab in a phase I clinical trial (**Table 1**). Therefore, it is essential to keep this limitation in mind and explore nanoparticle- or antibody-based delivery systems to achieve precision therapy and maximize therapeutic efficacy.

### 6.3 Interactions

#### 6.3.1 Checkpoints

Anti-PD1/PDL1 therapies have shown remarkable efficacy in multiple tumor types, but their effectiveness is largely limited to patients with high PD-L1 expression prior to treatment, leaving a significant portion of the patient population in need of alternative immune checkpoint inhibitors. Chronic inflammation can induce the expression of various immune checkpoint molecules, including PD1/PDL1, SLAMF7, CLEVER1, B7H4, and VISTA, which all have potential as therapeutic targets. For instance, the VISTA-PSGL1 axis has been extensively studied, and both anti-VISTA and anti-PSGL1 are currently being developed (85, 86, 326, 327). In a mouse model of lung cancer, an anti-Clever-1 antibody demonstrated superior performance to anti-PD-1 antibodies, reducing tumor growth in a TNBC model that is resistant to anti-PD-1 treatment (328). Phase I/II trials (NCT03733990) in patients with advanced solid tumors further demonstrated safety, tolerance, and preliminary immunostimulatory and antitumor activity (329, 330).

Phagocytosis and antigen uptake by TAMs are essential for subsequent immune stimulation. Targeting the “don’t-eat-me” signal CD47 and its receptor, SIRPa, enhances phagocytosis and antigen presentation by TAMs (331, 332 NCT02953782). Dual recognition antibodies are being investigated to improve specificity and avoid off-target effects; for example, anti-CD47&CD20 treatment is under clinical investigation in B-cell lymphoma (NCT03804996). LILRB on TAMs binds to MHC-I on cancer cells and inhibits macrophage phagocytosis. LILRB is reported to mediate resistance to various immunotherapies, and anti-LILRB1 (MK-4830) has demonstrated promising antitumor effects, along with a potent ability to reprogram TAMs and increase CD8+ T cells, as monotherapy or in combination with anti-CD47 or pembrolizumab (333, NCT03564691). Anti-SIGLEC10-mAb prevents the interaction between SIGLEC10 and another “don’t-eat-me” signal CD24, improving TAMs’ phagocytosis of tumor cells (334).

#### 6.3.2 Stimulatory

As discussed in detail in previous sections of this review, co-stimulatory interactions between TAMs and T cells are indispensable for effective antitumor immunity. Among the co-stimulatory molecules investigated, CD40, OX40, ICOS, and 4-1BB have been extensively studied (335).

The development of CD40 agonists has experience diverse molecular modification and optimization, evolving from CD40L-like structures to agonistic antibodies. However, most of these available agents demonstrated acceptable adverse effects but limited antitumor responses in monotherapy (336). Selicrelumab is an exception with promising therapeutic effects, reported to achieved a PR of 27% in patients with advanced melanoma (337). Combination with chemotherapeutic agents, other mAbs against PD-1, PD-L1, Flt3L, and VEGF, and MEKi are under active clinical investigation (32, 338–341). Interestingly, the combination of anti-CSF-1R antibody and agonistic anti-CD40 antibody transient TAM hyperpolarization and subsequently T cell activation before final depleting effect, emphasizing the importance of time (342, 343).

OX40 agonist preferentially drives M1 polarization over M1 (344, 345). A study in the Pan02 model of mouse pancreatic cancer revealed that the combination of agnostic anti-OX40 and inhibitory anti-CTLA4 led to transient decrease in ARG1 expression in TAMs, giving a therapeutic window for gemcitabine (346). However, other voices have suggested that OX40 agonist therapy actually increase ARG1 in TAM, justifying its combination with ARG1 inhibitor to improve efficacy (347). Additionally, the pro-inflammatory effects of OX40 agonist is further enhanced by Gal-3 inhibitior (belapectin) (348).

ICOS/ICOSL has dual roles with both pro- and anti-tumor activities, leading to T cell activation but also Treg differentiation, justifying the development of both anti-ICOS agonists (GSK3359609, JTX-2011) (NCT04428333, NCT02904226) and antagonists (MEDI-570, KY1044/Alomfilimab/SAR445256) (NCT02520791, NCT03829501). INDUCE-1 trial (NCT02723955) of agonist anti-ICOS-mAb in monotherapy or in combination with Pembrolizumab in patients with advanced solid tumors reported promising tolerability and antitumor activity (349). As patients treated with anti-CTLA-4 or anti-PD-1 had expanded the ICOS+FoxP3^+^ T cells, which are reported to be an important biomarker for clinical response, suggesting optimal response with combined therapies (350, 351). However, ICOS-ICOSL has also been considered immunosuppressive, mediates repair processes in liver damage (352) and skin wounding (61, 353), mechanistically through induction of Th2 cytokines (IL-4, IL-6, IL-10), M2 (61) and Tregs (354), leading to fever in developing anti-ICOSL immunotherapies (354, 355). Antagonistic anti-ICOS-mAbs had limited antitumor activity (356) but anti-inflammatory wound healing effects (61).

Intratumoral administration of 4-1BB agonistic Ab led to increased T cell infiltration not only through T and NK cell activation (65, 357, 358), but also activating effects in TAMs (359).

### 6.4 Cellular therapy

T cell-based cellular therapies have demonstrated success in treating hematogenous tumors but not solid tumors. Macrophage-based cellular therapy is a promising approach due to their superior ability to infiltrate into the hostile TME of solid tumors and potent secretory capacity. Similar to CAR-T, macrophages can also be loaded with a CAR construct against specific antigens. However, instead of direct cytotoxicity, CAR-M relies primarily on phagocytosis and antigen presentation to modulate the TME rather than directly eliminating tumor cells (360). As drivers of inflammation, CAR-M can synchronize an amplified anti-tumor immune response, transforming an immunologically cold TME into an inflamed battlefield.

Advancements in macrophage-related technologies have largely overcome their inherent resistance to expansion and genetic manipulation (361). Induction of macrophage differentiation from iPSC allowed for efficient *in vitro* amplification, making large-scale production for clinical application possible (362). CAR-M has demonstrated promising results in preclinical studies with cellular and animal models. Anti-HER2-CAR-M was safe and tolerable with optimistic therapeutic effects in early clinical trials (NCT04660929).

CAR-Ms, similar to endogenous macrophages, are also dynamically polarized to display pro- or anti-inflammatory functions and phenotypes. Combination with other macrophage polarization methods help prevent immunosuppressive polarization with pro-tumor effects. Adoptive cellular transfer has extra advantages as *in vitro* amplification provides a chance for precise genetic manipulation. Chimeric vector with co-stimulatory domains, engineered constitutive expression of IFNa (363, 364, NCT03866109), and knockout of pro-M2 genes are potential methods to maintain the desired M1 phenotype, coming into clinical testing. Similar to fourth-generation CAR-T, or TRUNKS, CAR-M can also be loaded with cytokines such as IL-12 to amplify type 1 immune response (365), or even with drug-containing nanoparticles to take advantage of their superior efficiency in tumor homing and improve drug delivery (366).

Furthermore, macrophages not only serve as tools of cellular therapy but also as targets. Despite challenges due to the heterogeneity of TAMs, CAR-Ts have the potential to specifically deplete detrimental TAMs while sparing inflammatory TAMs needed for antitumor immunity. Anti-F4/80 CAR-T in a mouse tumor model unselectively depleted all TAMs (367), while anti-FRb CAR-T, an M2 marker, allowed for specific targeting of immunosuppressive TAMs and restrained tumor growth (368).

## 7 Perspective

Immunotherapy revolutionized the clinical courses of tumor treatment, prolonging survival and offering patients with previously considered unresectable tumors a chance at surgery. However, only selected tumor types and a limited population of patients are responsive to anti-PD1/PDL1 antibodies, largely due to the hostile, immunosuppressive microenvironment. Much effort has been devoted to elucidating the mechanism of resistance and subsequently developing interventions, all leading to the importance of tumor immunogenicity and local inflammation.

Inflammation has a dual role in antitumor immunity, complicated by spatial-temporal factors and immune cells under influence. This double-edged sword is indispensable for the priming and activation of anti-tumor immunity, but also responsible for exhaustion and reactive desmoplasia. TAMs not only are the major cellular population of the TME, but also have superior secretory capacities. As the master regulators of TME inflammation, TAMs are capable of initiating and fine-tuning the cascade amplification of immune response, making them valuable targets. However, TAMs are highly heterogeneous and dynamic, existing in an almost inseparable spectrum of statuses, rather than the distinct M1/2 characteristics defined by *in vitro* stimulation or a state of terminal differentiation. TAMs are constantly affected by environmental factors including metabolic availability, stress, and cellular crosstalk through both direct contact and secreted factors. Upon sensing these stimuli, activation of coupled signaling pathways leads to transcriptional and epigenetic reprogramming, ultimately achieving functional and phenotypical polarization.

Such complexity and plasticity offer many therapeutic opportunities but at the same time pose challenges for precise and effective targeting, especially in the translation from bioinformatic data mining to experimental validation and further therapeutic strategy development. Still in the prime of its age, the development of TAM-centered therapies must adopt novel approaches to seek convergent points, striving to produce the butterfly effect in addition to specific targeting of each individual factor. In this review, we integrated recently published bioinformatic data, experimental studies, and advancements in clinical trials to provide a comprehensive understanding on the TAM polarization-inflammation process and potential therapeutic development. TAMs are promising tools to regulate TME inflammation, optimizing antitumor immune activation while minimizing protumor exhaustion and desmoplasia.

## Author contributions

JH and LD contributed equally to this work. All authors contributed to the article and approved the submitted version.
